# Hydrolytical instability of hydroxyanthraquinone glycosides in pressurized liquid extraction

**DOI:** 10.1007/s00216-014-7744-5

**Published:** 2014-03-22

**Authors:** Dorota Wianowska

**Affiliations:** Department of Chromatographic Methods, Faculty of Chemistry, Maria Curie-Sklodowska University, pl. Maria Curie-Sklodowska 3, 20-031 Lublin, Poland

**Keywords:** Hydroxyanthraquinones, Extraction, Hydrolytical instability, Glycosides, Aglycones, PLE

## Abstract

Hydroxyanthraquinones represent a group of pharmacologically active compounds characteristic for plants of the *Rumex* and *Rheum* genera. These compounds in the human intestine act as laxative compounds. As they cause the greatest side effects and are often abused by the public, their accurate analysis in plants and plant-derived laxatives is much needed. To isolate compounds from plants, pressurized liquid extraction (PLE) is frequently applied. The technique has been regarded, so far, as very effective, even in isolation of sensitive compounds for which exposure time in high temperature has a negative impact. This work demonstrates some interesting and surprising results accompanying PLE of hydroxyanthraquinones from the *Rumex crispus* L. root using methanol/water mixtures as extractant. The presented results demonstrate that glycoside forms of hydroxyanthraquinones (emodin*-*8*-O-β-D-*glucopyranoside, chrysophanol*-*8*-O-β-D-*glucopyranoside, and physcion*-*8*-O-β-D-*glucopyranoside) are hydrolytically unstable even in the short-lasting PLE. The increase of water concentration in the extractant leads to the increase of the transformation degree of the glycoside forms to the corresponding aglycones (emodin, chrysophanol, and physcion), increasing the concentration of the latter. The rise in the PLE temperature accelerates the hydrolytical degradation of the glycoside forms. The extension of the extraction time also intensifies this process. The presented results show that extraction of glycosides using extractants containing water can lead to false conclusions about the chemical composition of plants.

**Electronic supplementary material:**

The online version of this article (doi:10.1007/s00216-014-7744-5) contains supplementary material, which is available to authorized users.

**Figure:**
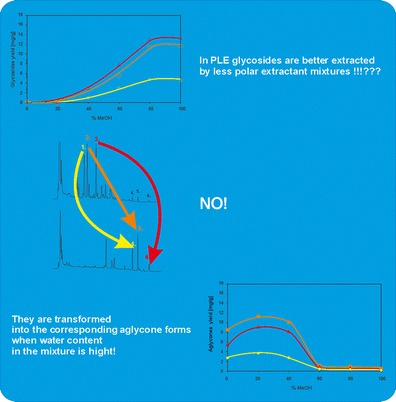
Hydrolytical instability of hydroxyanthraquinone glycosides even in the short-lasting PLE

## Introduction

Owing to the character and complexity of plant material, any analysis of plant constituents requires the application of an appropriate procedure of plant sample preparation, the aim of which is to extract the analyzed compounds from the matrix. Among a large number of sample preparation methods, solid–liquid extraction is most frequently used for the purpose. Much attention has been paid recently to the supported techniques of extraction, such as microwave-assisted solvent extraction, ultrasonic-assisted solvent extraction, supercritical fluid extraction, and pressurized liquid extraction (PLE). The latter technique enjoys a particularly great interest of researchers. This is due to the fact that PLE is regarded as a sample preparation technique allowing for a very quick and efficient extraction of the analyte from the plant matrix [1, 2]. Shortening the extraction process duration is particularly advantageous in the isolation of compounds for which exposure time in high temperatures is important. The fact that in PLE the plant sample is extracted in an inert atmosphere protected from light additionally decreases the risk of chemical degradation of extracted compounds and increases the attractiveness of its application. A number of studies have demonstrated the usefulness of the PLE technique for extraction of sensitive bioactive compounds, including hydroxyanthraquinones [1–3].

Hydroxyanthraquinones represent a group of pharmacologically active compounds characteristic for plants of the *Rumex* and *Rheum* genera [4, 5]. In these plants, hydroxyanthraquinones occur mostly as glycoside forms, mainly monoglucosides. Aglycones constitute only a few percent of their total amount [6]. As glycoside and aglycone forms differ in physicochemical properties, different extractant compositions are applied for their isolation [3, 7–10]. Aglycone forms, which are less polar than their glycoside forms, are better soluble in methanol and in water mixtures containing its significant concentration. Methanol/water mixtures rich in water, in turn, are recommended for the extraction of glycosides [7–10]. It must be remembered that the glycosides stability, in extraction conditions, is different and depends on chemical structure, temperature, pH, light, oxygen, and solvent type [11–14]. The problem of the hydrolytical stability of glycosides, however, is rarely taken into consideration during plants analysis. In the case of hydroxyanthraquinone glycosides, the problem of their accurate analysis in plants and plant-derived diet supplements is of particular importance. This is because hydroxyanthraquinones as laxative compounds are often abused by the public and glycosides, as more easily absorbed by the human body, cause the greatest side effects [3, 8, 15].

The paper discusses the hydrolytical stability of glycoside forms of hydroxyanthraqionones during their extraction from *Rumex crispus* root in different PLE conditions using methanol/water mixture as extractant. During the experiments, the concentration changes of the chosen monoglycosides (emodin*-*8*-O-β-D-*glucopyranoside, chrysophanol*-*8*-O-β-D-*glucopyranoside, and physcion*-*8*-O-β-D-*glucopyranoside) and their aglycones (emodin, chrysophanol, and physcion) were examined. As parameters affecting the hydrolitycal stability of the glycosides in the PLE process different compositions of metanol/water mixture, extraction temperatures, pressures, and static extraction times were investigated.

## Materials and methods

### Plant material and chemicals

Plant material was collected in autumn time in the Botanical Garden of Maria Curie Sklodowska University, Lublin, Poland. The root of *R. crispus* L. was air-dried at room temperature for 4 weeks (moisture content 9.98 %, determined by drying in an oven at 105 °C for 2 h). A sufficiently large representative sample of the material was ground into powder. The powder was screened with a sieve and particle sizes, between 40 and 70 mesh, were used for this study (~0.3 mm). Precisely weighted portions of the powdered root were used to test hydrolytical instability of chosen hydroxyanthraquinones in PLE conditions. Standards of hydroxyanthraquinone aglycones—emodin, chrysophanol, and physcion—were purchased from Sigma-Aldrich, Poland. Emodin*-*8*-O-β-D-*glucopyranoside, chrysophanol*-*8*-O-β-D-*glucopyranoside, and physcion*-*8*-O-β-D-*glucopyranoside, isolated from the powdered *Rheum palmatum* root following the procedure described in [16], were used as hydroxyanthraquinone monoglucosides standards. The structures of these compounds were identified by comparing their physicochemical and spectral data with those published in the literature [9, 10, 17–19]. The purity of the compounds was above 95 % as confirmed by high-performance liquid chromatography (HPLC) analysis. Methanol (HPLC and analytical-reagent grade) and glacial acetic acid (analytical-reagent grade) were purchased from POCh (Gliwice, Poland). Water, purified on a Milli-Q system from Millipore (Millipore, Bedford, MA, USA), was used throughout the experiments. Neutral glass, obtained as a gift from local glassworks (fraction, 0.4–0.6 mm) was applied as a dispersing agent in the PLE extraction cell.

### HPLC analysis

HPLC measurements were performed on a Dionex liquid chromatograph (Dionex Corp., Sunnyvale, CA, USA) consisting of a chromatography enclosure (LC20) equipped with a Rheodyne automated injection valve with a 25-μL sample loop attached, a gradient pump (GP50), an absorbance detector (AD25), and a photodiode array detector (PDA100). All the analyses were under control of the PeakNet6 data acquisition system. Chromatographic separations were carried out using a Prodigy ODS-3 column (5 μm, 250 × 4.6 mm I.D.; Phenomenex, Torrance, CA, USA) and a security guard column of the same producer. The column and the guard column were placed in the oven and the analyses were performed at 30 °C (Column Thermostat, JetStream II Plus, Knauer, Warsaw, Poland). The chromatographic analyses were realized in gradient elution conditions at a flow of 1 mL/min using aqueous acetic acid solution (0.5 mL of glacial acetic acid in 100 mL of the solution) as solvent A, and methanol as solvent B. The following gradient program of the mobile phase was applied: linear increase of B from 45 to 85 % (0–30 min), next 85 % B during 10 min, and finally linear decrease of B from 85 to 45 % during 2 min. At the end, the column was conditioned using the mobile phase containing 45 % B (5 min). Each extract was HPLC analyzed three times. The chromatograms were monitored by the AD25 detector at 254 nm and by the PDA detector. The absorbance spectra (190–750 nm) were collected continuously in the course of each run.

The concentrations of the respective analytes in the resulting extracts were calculated from the calibration curves obtained separately for each compound in the concentration range 2.5–100.0 μg/mL. Each calibration curve was generated from five concentrations of the compound. Three measurements of peak area for each concentration of standard solution were performed. Table S1 (see Electronic supplementary material) presents the characteristic parameters of the calibration curves, where *a* and *b* are coefficients of equation regression *y = ax + b*, and *r* ^2^ is the correlation coefficient of the equation. As shown in Table S1, the calibration curves were found to be linear in the tested concentration range. The correlation coefficient was found to be > 0.997 for all the examined compounds. The limit of detection was within the range 0.0623–0.1624 μg/mL.

The RSDs of the retention times and peak areas of the examined hydroxyanthraquinones in the methanolic extract from the *R. crispus* root obtained by PLE were taken as the measurements of the method reliability (*n* = 6). As shown in Table S2 (see Electronic supplementary material), the overall RSD values were less than 2.60 %, indicating that the developed method is satisfactory for the hydroxyanthraquinones analysis in *R. crispus* root.

### HPLC/ESI/MS analysis

To confirm the identification of the examined compounds, HPLC/ESI/MS analyses were performed. The analyses were carried out on a Dionex HPLC instrument connected to a Finnigan ion-trap mass spectrometer (ThermoElectron Corp., San Jose, CA) via an electrospray ionization (ESI) source. The column and elution conditions used were the same as those used in HPLC analysis except that the flow rate was set at 0.5 mL/min. Ultrahigh-purity helium was used as the collision gas and high-purity nitrogen (N_2_) as the nebulizing gas. The optimized mass spectrometry detector parameters in the negative ion mode were as follows: ion spray voltage, 4.5 kV; sheath gas (N_2_), 60 arbitrary units; auxiliary gas (N_2_), 15 units; capillary temperature, 350 °C; capillary voltage, −15 V; and tube lens offset voltage, −10 V. For full-scan MS analysis, the spectra were recorded in the range *m/z* 100–1,000. To identify the investigated compounds, the functions of secondary (MS^*n*^) ion fragmentation were applied. The collision-induced dissociation energy was chosen individually for each examined compound.

### Sample preparation

#### Extraction under reflux

A weighted portion of the *R. crispus* root (2.0 g) was heated for 30 min under a reflux condenser at the boiling temperature of methanol. After cooling, the extract was removed and a new portion of fresh extractant was added to the remaining material. The process was repeated three times. All the extracts were pooled together into a 200-mL volumetric flask and filled up to its volume with methanol. Extractions under reflux were repeated three times on fresh portions of the material. The extracts were subjected to the HPLC analysis.

#### Pressurized liquid extraction

PLE was performed with a Dionex ASE200 instrument (Dionex Corp., Sunnyvale, CA, USA). To reduce the volume of the solvent used for the extraction [20], the *R. crispus* samples (0.5 g), or chrysophanol*-*8*-O-β-D-*glucopyranoside (0.1 mg), were mixed with neutral glass and placed into a 22-mL stainless steel extraction cell. The conditions of the PLE procedure (extractant composition, temperature, pressure and time) are given in the “Results and discussion.” Depending on the packing density of the extraction cells, the volume of the collected extracts was between 25 and 31 mL. Between the runs, the system was washed with the extractant. The obtained extracts were transferred to 50 mL volumetric flasks, filled up to their volume with extractant, and subjected to the HPLC analysis.

For statistical purposes, each sample preparation procedure was repeated three times in identical experimental conditions.

The extraction efficiency for PLE was determined by performing consecutive PLE on the same sample, until no investigated compounds were detected by the analysis. These experiments were performed in conditions recommended as default by Dionex Corporation (i.e., 100 °C, 10 min, 60 bar), using methanol. The efficiency was calculated based on the total amount of individual investigated compounds. The recoveries at one-time extraction obtained for every analyte were higher than 98.5 % (RSD < 7 %, *n* = 5). The obtained results are in good agreement with the literature data [3].

### Statistical analysis

All data are expressed as mean ± standard deviation. The analysis of variance (ANOVA) and *F* test were used to assess the influence of experimental conditions on PLE yield. The mean values were considered significantly different when result of compared parameters differed at *P* = 0.05 significance level. To check the significance of each Fisher coefficient the *P* values were used. For all statistics Microsoft Excel™ 2010 was used.

## Results and discussion

**Fig. 1:**
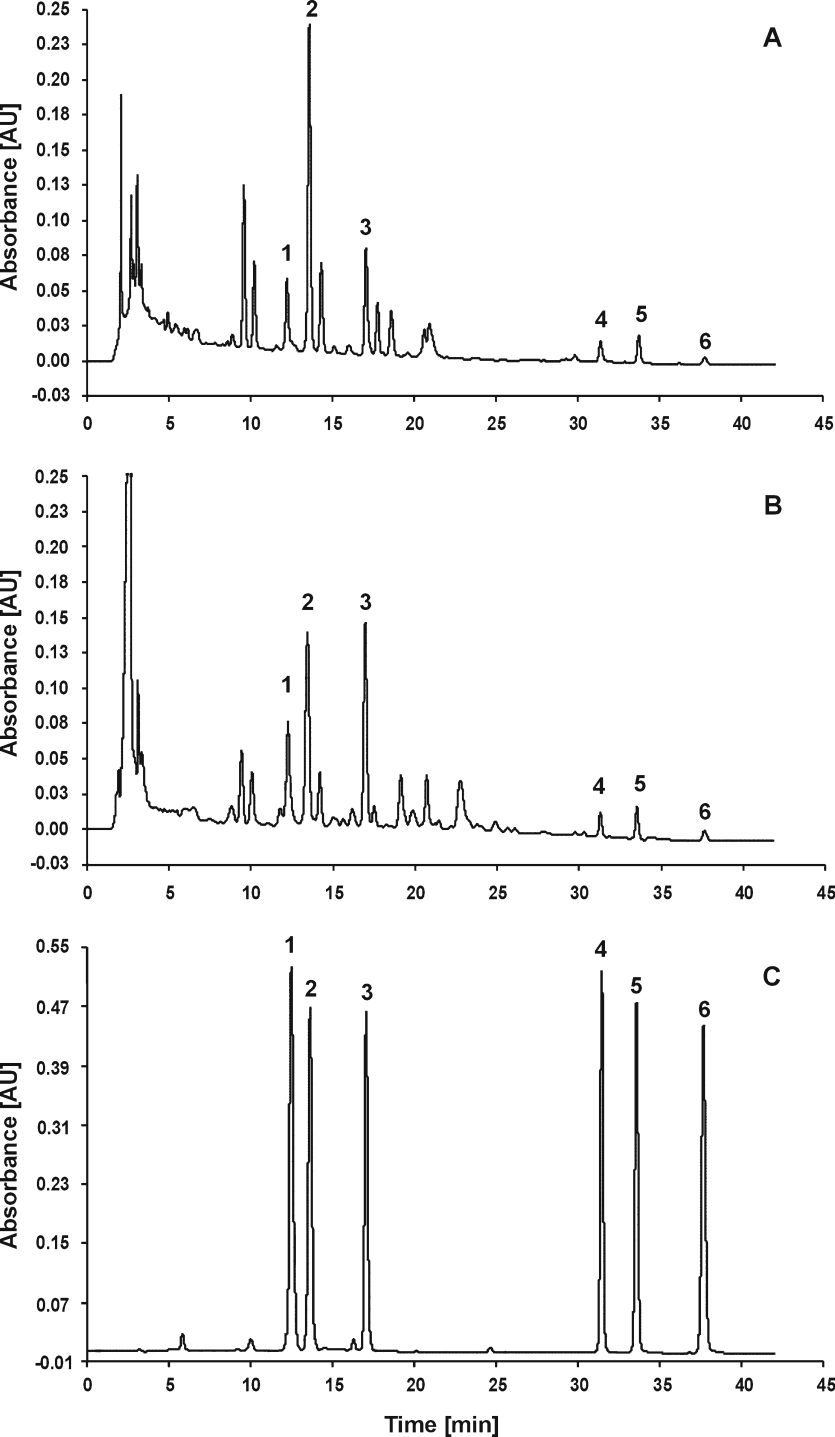
Exemplary chromatograms of methanolic extracts from the *R. crispus* L. root obtained by reflux extraction (**a**) and PLE (**b**). **c** The chromatogram of standard mixture of the examined hydroxyanthraquinones. *Peaks 1*, emodin*-*8*-O-β-D-*glucopyranoside; *2*, chrysophanol*-*8*-O-β-D-*glucopyranoside; *3*, physcion*-*8*-O-β-D-*glucopyranoside; *4*, emodin; *5*, chrysophanol, and *6*, physcion

The exemplary chromatograms of methanolic extracts from the *R. crispus* root obtained by extraction under reflux and PLE, at methanol boiling point, are presented in Fig 1a, b, respectively. Figure 1c presents the chromatogram of standards mixture of the examined hydroxyanthraquinones. As results from the figure, the applied chromatographic conditions allow for a sufficient resolution of the examined compounds from sample matrix peaks. In the chromatograms of extracts, the peak identity and purity were determined on the basis of their chromatographic behaviors, UV–vis spectra and characteristic mass spectrometric fragmentation features. High-performance liquid chromatography/photodiode array detector/electrospray ionization/mass spectrometry (HPLC/PAD/ESI/MS^*n*^) data identification of the examined compounds are summarized in Table S3 (see Electronic supplementary material). The chromatograms in Fig. 1a, b show that the PLE extract composition is very similar to that obtained using extraction under reflux, which is the most commonly used technique of hydroxyanthraquinones extraction from plants.

**Fig. 2:**
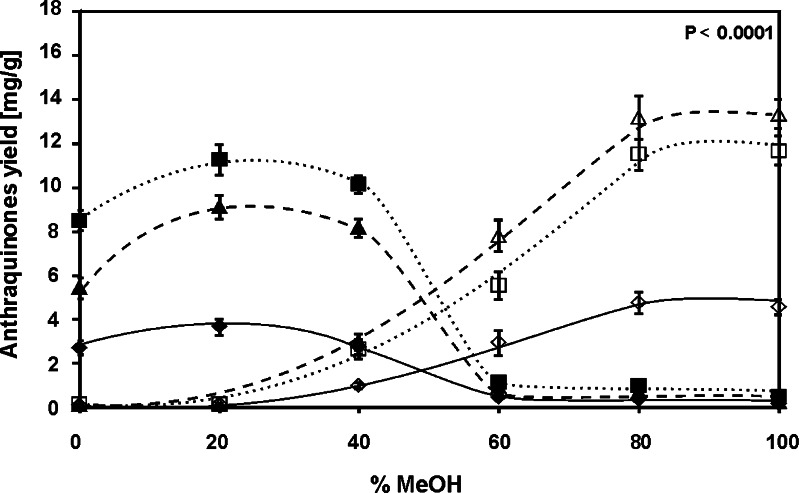
The influence of the composition of methanol/water mixture used as the PLE extractant on the extraction yields of examined hydroxyanthraquinones of the *R. crispus* root: emodin*-*8*-O-β-D-*glucopyranoside (*white diamonds with solid line*), chrysophanol*-*8*-O-β-D-*glucopyranoside (*white squares with dotted line*), physcion*-*8*-O-β-D-*glucopyranoside (*white triangles with dashed line*), emodin (*black diamonds with solid line*), chrysophanol (*black squares with dotted line*), and physcion (*black triangles with dashed line*). PLE conditions: temperature, 100 °C; time, 10 min; and pressure, 40 bar (*n* = 3)

PLE is one of the very few techniques of solids extraction which allows to easily enhance the extraction selectivity using multi-component extractants. Figure 2 shows the influence of the composition of methanol/water mixture on the extraction yields of the examined hydroxyanthraquinones of the *R. crispus* root. The importance of the experimental factors determined according to the *F* value for the individual compounds is listed in Table S4 (see Electronic supplementary material). The analysis of the presented in Fig. 2 relationships leads to the conclusion that the extraction efficiency of aglycones, which are less polar compounds than their glycoside forms, is better in more polar methanol/water mixtures than in less polar methanol. The examined glycosides are better extracted by methanol and methanol/water mixtures containing a small amount of water. The results presented in Fig. 2 may seem surprising as the opposite extraction ability of methanol and methanol/water mixtures for aglycones and their glycoside forms is expected (see “Introduction”). However, the obtained results can be explained by the hydrolysis of glycosides to the corresponding aglycones.

**Fig. 3:**
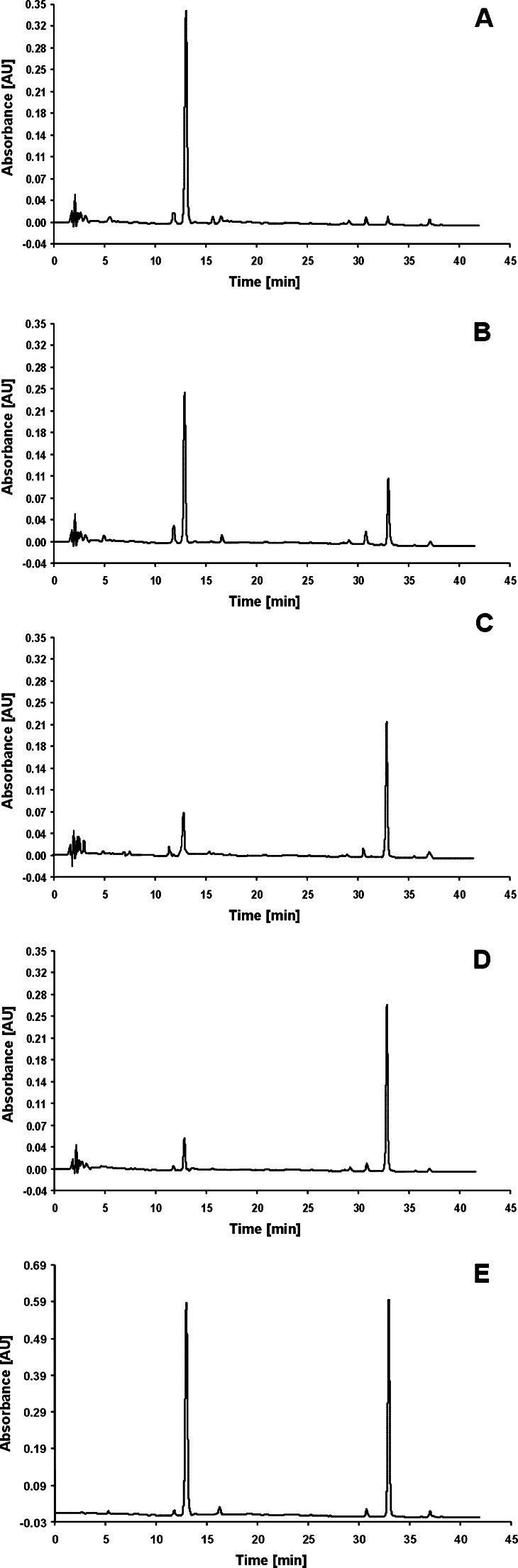
The chromatograms of chrysophanol*-*8*-O-β-D-*glucopyranoside extracted from PLE cell using: **a** methanol; **b** methanol/water mixture 80/20 (*v*/*v*); **c** methanol/water mixture 40/60 (*v*/*v*), and **d** methanol/phosphoric buffer, pH = 3.05 mixture 80/20 (*v*/*v*). PLE conditions: temperature, 100 °C; time 10 min and pressure, 40 bar. **e** The chromatogram of standards mixture of chrysophanol*-*8*-O-β-D-*glucopyranoside and of chrysophanol

As appears from the literature [11–14], the stability of glycosides is different. However, the problem of their hydrolytic stability during extraction process, especially in short-lasting PLE, is rarely taken into consideration during plants analysis. In the light of the results shown in Fig. 2, it was decided to establish if glycoside forms of hydroxyanthraquinones were hydrolytically stabile in PLE. To check their hydrolytic stability, the PLE process of a chosen glycoside form of hydroxyanthraquinones was simulated. Instead of plant material, chrysophanol*-*8*-O-β-D-*glucopyranoside standard was placed in the PLE cell. The results are presented in Fig. 3. These data were obtained applying four types of extractants: methanol (see Fig. 3a); methanol/water mixture, 80/20 (*v*/*v*) (see Fig. 3b); methanol/water mixture, 40/60 (*v*/*v*; see Fig. 3c), and methanol/phosphoric buffer mixture, 80/20 (*v*/*v*), pH = 3.05 (see Fig. 3d). As appears from the presented data, a 10-min PLE process with methanol/water mixture leads to the appearance of chrysophanol in the extract. Moreover, the amount of this aglycone increases when water concentration in methanol/water extractant is greater (cf. Fig. 3b, c). The results from Fig. 3 did not only demonstrate that the glycoside forms of hydroxyanthraquinones are hydrolytically unstable in PLE but also explain the shape of the relationships in Fig. 2. The increase of water concentration in methanol/water extractant accelerates the hydrolytic degradation of the glycoside form of hydroxyanthraquinones (113.97 < *F* < 335.98, *F*_crit_ = 3.1059, Table S4, see Electronic supplementary material). This process is responsible for the increase of aglycone concentration in extracts containing higher concentration of water (115.52 < *F* < 515.07, *F*_crit_ = 3.1059). The chromatogram in Fig. 3a shows that a 10 min PLE with methanol does not lead to a visible decomposition of chrysophanol*-*8*-O-β-D-*glucopyranoside. Its hydrolysis is enhanced by the presence of hydrogen ions in the extractant (cf. Fig. 3b, d). It is worth noticing that the plants of the *Rumex* species contain a significant amount of oxalic acid, which is responsible for the acidic character of extracts and probably facilitates the hydrolysis of the glycoside forms of hydroxyanthraquinones [21].

**Fig. 4:**
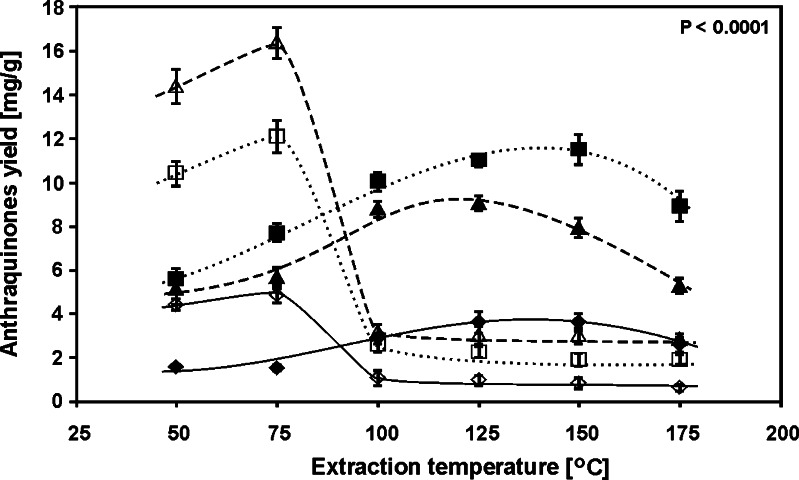
The influence of PLE temperature on the extraction yields of examined hydroxyanthraquinones of the *R. crispus* root: emodin*-*8*-O-β-D-*glucopyranoside (*white diamonds with solid line*), chrysophanol*-*8*-O-β-D-*glucopyranoside (*white squares with dotted line*), physcion*-*8*-O-β-D-*glucopyranoside (*white triangles with dashed line*), emodin (*black diamonds with solid line*), chrysophanol (*black squares with dotted line*), and physcion (*black triangles with dashed line*). PLE conditions: methanol/water mixture, 40/60 (*v*/*v*); time, 10 min; and pressure, 40 bar (*n* = 3)

One of the main factors influencing extraction efficiency of compounds from plants matrices is temperature. Temperature, however, may also be involved in the hydrolytic decomposition of glycosides, when methanol/water extractants are used, changing the kinetics of the process. Therefore, in the next step of investigations it was decided to check how the extraction temperature change alters the quantitative relationships between the glycoside and aglycone forms of hydroxyanthraquinones. The experiments were carried out using such composition of the extraction mixture at which high concentration of both forms of hydroxyanthraquinones is observed (i.e., methanol/water mixture, 40/60, *v*/*v*). Figure 4 illustrates the influence of extraction temperature on the yield of hydroxyanthraquinones when methanol/water mixture (40/60, *v*/*v*) is used. In the temperature range 50–150 °C, a gradual concentration increase of all the examined aglycones with temperature increase is observed. The decrease of aglycones concentration in the extracts obtained above 150 °C can be attributed to their thermal decomposition. The influence of extraction temperature on the concentration of glycoside forms is different. In the temperature range 50–75 °C, a small increase of their concentration with temperature increase is observed. The concentration of glycosides decreases rapidly above 75 °C, whereas the concentration of individual glycosides in the extracts obtained above 100 °C is almost constant. As it was shown above, glycoside forms of hydroxyanthraquinones are hydrolytically unstable. Thus, the relationships presented in Fig. 4 can be treated as an overlap of a few factors: the influence of the temperature rise on the change of the extraction efficiency, on the kinetic of glycoside hydrolysis and on kinetic of aglycone degradation. For the applied extractant composition, the increase of extraction temperature above 75 °C apparently accelerates the hydrolytic degradation of the glycoside forms of hydroxyanthraquinones (161.92 < *F* < 466.43, *F*_crit_ = 3.1059, see Table S4).

**Fig. 5:**
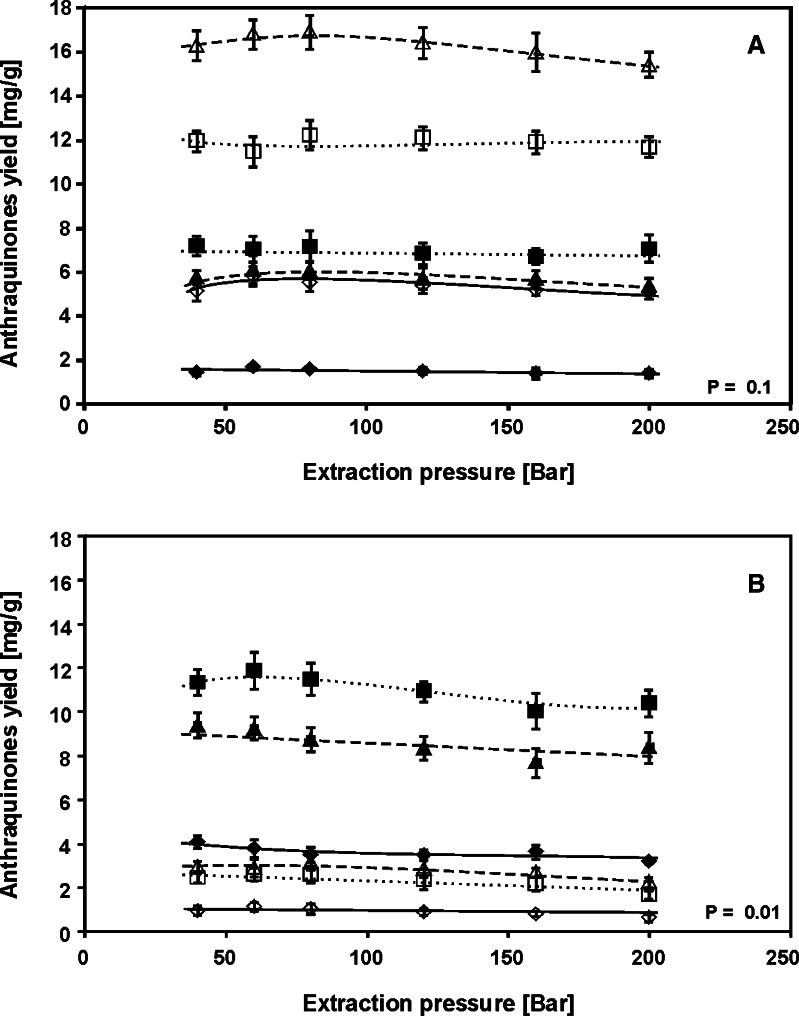
The influence of PLE pressure on the extraction yield of the examined hydroxyanthraquinones of the *R. crispus* root at 75 (**a**) and 125 °C (**b**). For symbols, see Fig. 4. PLE conditions: methanol/water mixture, 40/60 (*v*/*v*) and time, 10 min (*n* = 3)

The influence of extraction pressure increase in the range 40–200 bar on the extraction yields of the examined hydroxyanthraquinones obtained using methanol/water mixture (40/60, *v*/*v*) at 75 and 125 °C is presented in Fig. 5a, b, respectively. As appears from the data, the effect of extraction pressure on the concentration change of the compounds is negligible. The *F* value (see Table S4) confirms that the differences between the yields are statistically insignificant (*F*_exp_ ≤ *F*_crit_).

**Fig. 6:**
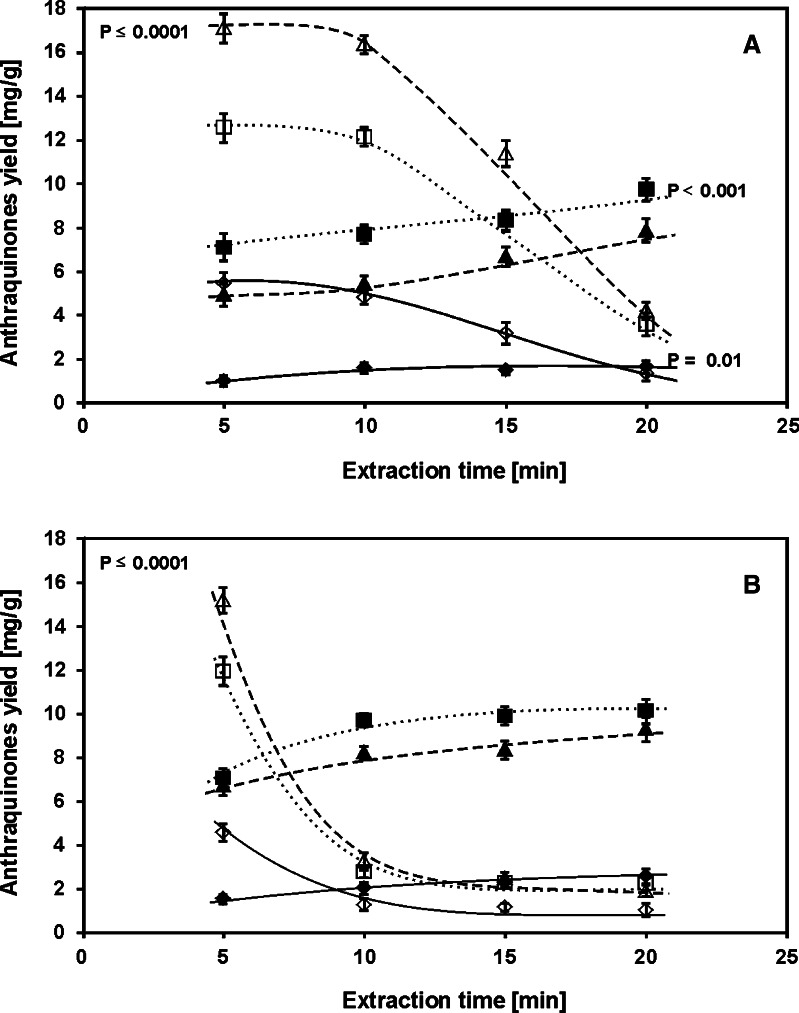
The influence of PLE time on the extraction yields of examined hydroxyanthraquinones of the *R. crispus* root at 75 (**a**) and 125 °C (**b**). For symbols, see Fig. 4. PLE conditions: methanol/water mixture, 40/60 (*v*/*v*) and pressure, 40 bar (*n* = 3)

The results presented so far were obtained using a 10 min PLE process. The effect of extraction time increase in the time range 5–20 min on the concentration of the examined hydroxyanthraquinones in methanol/water extractant (40/60, *v*/*v*) at 75 and 125 °C is presented in Fig. 6a, b, respectively. As results from the figure, the extension of the extraction time intensifies the hydrolytical degradation of the glycoside forms of hydroxyanthraquinones even at 75 °C, however, it becomes visible for longer extraction times (52.82 < *F* < 362.85, *F*_crit_ = 4.0662, see Table S4). The effect is more pronounced for the higher extraction temperature (93.41 < *F* < 753.52, *F*_crit*.*_ = 4.0662; see Fig. 6b). In this case, distinct changes of glycosides concentrations are seen between 5 and 10 min PLE. Although the extension of extraction time above 10 min does not lead to significant concentration changes of both forms of hydroxyanthraquinones, a very small concentration increase of aglycones is observed. The rapid concentration decrease of glycoside forms of hydroxyanthraquinones in extracts confirms their hydrolytic instability in PLE.

## Conclusions

Optimisation of the PLE process generally begins with the choice of an appropriate extraction solvent, with polarity close to that of the isolated compound. The extractant compositions giving the highest yield of the analyte are regarded as optimal. The fact that some compounds, during plant sample extraction, are transformed into others increasing their extraction yield is very often ignored. The results presented in this paper show that the glycoside forms of hydroxyanthraquinones (emodin*-*8*-O-β-D-*glucopyranoside, chrysophanol*-*8*-O-β-D-*glucopyranoside, and physcion*-*8*-O-β-D-*glucopyranoside) during their extraction from the *R. crispus* L*.* root are transformed to the corresponding aglycones (emodin, chrysophanol, and physcion) even in the short-lasting PLE when methanol/water mixtures are applied as extractant for their isolation. It was shown that the transformation results from the hydrolytical instability of glycosides. The increase of water concentration in methanol/water extractant mixture heightens the transformation degree of the glycoside forms to the corresponding aglycones increasing their concentration. The temperature growth accelerates this process. The extension of the extraction time, even at the lower extraction temperature, also enhances the process of hydrolytical degradation of the glycoside forms of hydroxyanthraquinones.

The presented results demonstrate that extraction of glycoside forms using extractants containing water can lead to false conclusions in plant samples analysis (e.g., when examining the quantity of the individual hydroxyanthraquinones in plants during, their growth or their processing).

## Electronic supplementary material

Below is the link to the electronic supplementary material.

ESM 1 (PDF 191 kb)
