# Supplementary material for: Hydrolytical instability of hydroxyanthraquinone glycosides in pressurized liquid extraction
Source: Anal Bioanal Chem. 2014 Mar 22;406(13):3219–27. doi: 10.1007/s00216-014-7744-5 (PMC3992226; doi:10.1007/s00216-014-7744-5)
Supplement: Supplementary file 1 — (PDF 191 kb) [file 216_2014_7744_MOESM1_ESM.pdf]

Analytical and Bioanalytical Chemistry

Electronic Supplementary Material

Hydrolytical instability of hydroxyanthraquinone glycosides in pressurized liquid extraction

Dorota Wianowska

Table S1. Linearity studies and limit of detection (LOD) for the examined hydroxyanthraquinones tested in HPLC chromatograms

**Analyte**	**Slope** *a*	**Intercept** *b*	r^2	**LOD*** **[µg/mL]**
Emodin-8-*O*-*β*-*D*-glucopyranoside	1.06060	0.00150	0.99926	0.12156
Chrysophanol-8-*O*-*β*-*D*-glucopyranoside	0.92696	0.00205	0.99949	0.14780
Physcion-8-*O*-*β*-*D*-glucopyranoside	0.86597	0.00145	0.99923	0.16239
Emodin	2.30375	0.00127	0.99991	0.06228
Chrysophanol	1.26580	0.00312	0.99785	0.07542
Physcion	1.22784	0.00162	0.99919	0.08357

* Calculated as three times of the signal-to-noise ratio.

Table S2. Determination of the reliability of the described HPLC method for six constituents of *Rumex crispus* L. (n = 6)

Compound	Intra-day RSD [%]		Inter-day RSD [%]	
	t_R*	PA**	t_R*	PA**
Emodin-8-*O*-*β*-*D*-glucopyranoside	0.68	1.26	1.24	1.77
Chrysophanol-8-*O*-*β*-*D*-glucopyranoside	0.52	1.43	0.98	1.15
Physcion-8-*O*-*β*-*D*-glucopyranoside	0.47	0.88	2.14	2.57
Emodin	0.23	0.62	0.66	1.22
Chrysophanol	0.19	0.65	0.57	0.98
Physcion	0.16	0.50	0.64	0.78

* Refers to retention time

** Refers to peak area

Table S3. HPLC/PAD/ESI/MS^n data identification of the examined hydroxyanthraquinones

**Compound no. from Figure 1**	**Compound name**	**λ_{max} [nm]**	**$[M-H]^-$ *m/z***	**MS^2 *m/z* (% intensity)**
**1**	Emodin-8-*O*-*β*-*D*-glucopyranoside	282, 424	431	311(15), 293(25), 269(90), 241(20), 225(60), 197(10)
**2**	Chrysophanol-8-*O*-*β*-*D*-glucopyranoside	282,424	415	325(10), 295(13), 277(15), 253(100), 238(10), 225(50), 210(6)
**3**	Physcion-8-*O*-*β*-*D*-glucopyranoside	282, 420	445	283(100), 268 (40), 253 (10), 240(60)
**4**	Emodin	284, 436	269	241(16), 225(100), 197(20)
**5**	Chrysophanol	254, 427	253	238(30), 225(100), 210(15)
**6**	Physcion	282, 434	283	268(100), 240(60), 212(10)

Table S4. *F*-values and *P*-values obtained during variance analysis for the effects of PLE conditions on the extraction yield of emodin-8-*O*-*β*-*D*-glucopiranoside (EG), chrysophanol-8-*O*-*β*-*D*-glucopyranoside (ChG), physcion-8-*O*-*β*-*D*-glucopyranoside (PG), emodin (E), chrysophanol (Ch), and physcion (P)

Compd	*Extractant compos.**		*Temperature**		*Pressure at 75 ^0C**		*Pressure at 125 ^0C**		*Time at 75 ^0C***		*Time at 125 ^0C***	
	F-value	*P*-value	*F*-value	*P*-value	*F*-value	*P*-value	*F*-value	*P*-value	*F*-value	*P*-value	*F*-value	*P*-value
EG	113.97	$1.10 \cdot 10^{-09}$	161.92	$1.40 \cdot 10^{-10}$	1.69	$2.11 \cdot 10^{-01}$	2.84	$6.41 \cdot 10^{-02}$	52.82	$1.29 \cdot 10^{-05}$	93.41	$1.44 \cdot 10^{-06}$
ChG	335.98	$1.85 \cdot 10^{-12}$	358.10	$1.27 \cdot 10^{-12}$	0.69	$6.39 \cdot 10^{-01}$	3.58	$3.24 \cdot 10^{-02}$	195.63	$8.00 \cdot 10^{-08}$	437.28	$3.31 \cdot 10^{-09}$
PG	331.52	$2.00 \cdot 10^{-12}$	466.43	$2.62 \cdot 10^{-13}$	1.80	$1.88 \cdot 10^{-01}$	3.05	$5.27 \cdot 10^{-02}$	362.84	$6.95 \cdot 10^{-09}$	753.52	$3.80 \cdot 10^{-10}$
E	115.52	$1.02 \cdot 10^{-09}$	42.50	$3.19 \cdot 10^{-07}$	1.35	$3.08 \cdot 10^{-01}$	3.26	$4.34 \cdot 10^{-02}$	4.65	$3.66 \cdot 10^{-02}$	7.89	$8.96 \cdot 10^{-03}$
Ch	515.07	$1.45 \cdot 10^{-13}$	55.28	$7.20 \cdot 10^{-08}$	0.35	$8.75 \cdot 10^{-01}$	3.14	$4.83 \cdot 10^{-02}$	14.51	$1.34 \cdot 10^{-03}$	31.41	$8.93 \cdot 10^{-05}$
P	378.48	$9.10 \cdot 10^{-13}$	46.62	$1.89 \cdot 10^{-07}$	1.10	$4.09 \cdot 10^{-01}$	3.40	$3.83 \cdot 10^{-02}$	23.92	$2.39 \cdot 10^{-04}$	17.47	$7.15 \cdot 10^{-04}$

* $F_{crit.} = 3.106$

** $F_{crit.} = 4.0$
